# Small Extracellular Vesicle Enrichment of a Retrotransposon-Derived Double-Stranded RNA: A Means to Avoid Autoinflammation?

**DOI:** 10.3390/biomedicines9091136

**Published:** 2021-09-01

**Authors:** Marilou H. Barrios, Alexandra L. Garnham, Andrew D. Foers, Lesley Cheng-Sim, Seth L. Masters, Ken C. Pang

**Affiliations:** 1 Advanced Technology and Biology Division, Walter and Eliza Hall Institute of Medical Research, Parkville, VIC 3052, Australia; barrios.m@wehi.edu.au (M.H.B.); garnham.a@wehi.edu.au (A.L.G.); 2 Department of Medical Biology, The University of Melbourne, Parkville, VIC 3010, Australia; masters@wehi.edu.au; 3 Kennedy Institute of Rheumatology, University of Oxford, Oxford OX3 7FY, UK; andrew.foers@kennedy.ox.ac.uk; 4 La Trobe Institute for Molecular Science, La Trobe University, Bundoora, VIC 3083, Australia; L.Cheng@latrobe.edu.au; 5 Inflammation Division, Walter and Eliza Hall Institute of Medical Research, Parkville, VIC 3052, Australia; 6 Genetics Theme, Murdoch Children’s Research Institute, Parkville, VIC 3052, Australia; 7 Department of Adolescent Medicine, The Royal Children’s Hospital, Parkville, VIC 3052, Australia; 8 Department of Paediatrics, The University of Melbourne, Parkville, VIC 3052, Australia

**Keywords:** exosomes, extracellular vesicles, VL30, endogenous retrovirus, retrotransposon

## Abstract

Small extracellular vesicles (SEVs) such as exosomes are released by multiple cell types. Originally believed to be a mechanism for selectively removing unwanted cellular components, SEVs have received increased attention in recent years for their ability to mediate intercellular communication. Apart from proteins and lipids, SEVs contain RNAs, but how RNAs are selectively loaded into SEVs remains poorly understood. To address this question, we profiled SEV RNAs from mouse dendritic cells using RNA-Seq and identified a long noncoding RNA of retroviral origin, VL30, which is highly enriched (>200-fold) in SEVs compared to parental cells. Bioinformatic analysis revealed that exosome-enriched isoforms of VL30 RNA contain a repetitive 26-nucleotide motif. This repeated motif is itself efficiently incorporated into SEVs, suggesting the likelihood that it directly promotes SEV loading. RNA folding analyses indicate that the motif is likely to form a long double-stranded RNA hairpin and, consistent with this, its overexpression was associated with induction of a potent type I interferon response. Taken together, we propose that preferential loading into SEVs of the VL30 RNA containing this immunostimulatory motif enables cells to remove a potentially toxic RNA and avoid autoinflammation. In this way, the original notion of SEVs as a cellular garbage bin should not be entirely discounted.

## 1. Introduction

Small extracellular vesicles (SEVs) such as exosomes are extracellular, membrane-bound vesicles that originate from the multivesicular bodies of the cell’s endocytic compartment. Originally considered a mechanism by which cells excrete unwanted materials [1], SEVs have since been recognized for their ability to mediate intercellular communication and influence the fate of recipient cells via a selective cargo of proteins, lipids and RNAs. Consistent with this, SEVs have received substantial attention in recent years as a novel means of delivering therapeutic payloads into the body for the treatment of various diseases [2,3].

Using SEVs to deliver therapeutic RNAs is seen as a particularly promising strategy [4,5]. However, it remains unclear how best to load SEVs with particular RNAs of interest. In this regard, knowing the mechanisms by which endogenous RNAs are naturally shuttled into SEVs would be beneficial, but to date few studies have examined this topic. Koppers-Lalic et al. previously reported that miRNAs are not randomly incorporated into exosomes but that the addition of bases at the 3′ end of a miRNA influences its sorting into exosomes [6]. Specifically, they showed that miRNAs rich in uridines (Us) at the 3′ end were enriched in exosomes compared to cells, and conversely that miRNAs rich in adenines (As) at the 3′ end are more likely to be sorted into cells. Villaroya-Beltri et al. on the other hand reported that certain motifs govern the sorting of miRNAs into exosomes [7]. They found that the protein hnRNPA2B1 binds specific sequences in miRNAs and facilitates their loading into exosomes [8], while other RNA-binding proteins have also been implicated in the sorting of miRNAs into SEVs (reviewed in [9]). A small number of studies have examined longer RNAs. One of these claimed that the presence of a “molecular zipcode” in the 3’ UTR of mRNAs serves as a “docking site” for miR-1289 and results in an enrichment of the mRNAs in the multivesicular body [10]. However, this enrichment was only modest (two-fold) and the authors did not examine whether this zipcode sequence led to enrichment within exosomes themselves. Another study identified three motifs bioinformatically (ACCAGCCU, CAGUGAGC and UAAUCCCA) that are enriched in exosomal mRNAs and long noncoding RNAs (lncRNA) [11]. These motifs exhibit double-stranded stem-loop structures and were subsequently reported to be recognised by the exosomal proteins YB-1 and NSUN2 [12].

To better understand RNA loading into SEVs, we assessed the mRNA and long noncoding RNA content of dendritic cell (DC) SEVs, which are already being explored therapeutically in clinical trials [13]. Using next generation sequencing, we found that one of the most highly enriched SEV transcripts was VL30, an endogenous RNA derived from a retroviral element. The abundance of VL30 within SEVs was several hundred times higher than in the parental DCs, a result that was subsequently confirmed in multiple other cell types. Bioinformatic analysis revealed that SEV-enriched isoforms of VL30 RNA contain a repetitive motif whose secondary structure is strongly predicted to form an extended double stranded RNA (dsRNA) hairpin. Expression of this motif alone resulted in its being preferentially loaded into SEVs and induced a strong type I interferon response and cell death. Taken together, we speculate that the preferential SEV loading of VL30 RNAs containing this dsRNA motif is a means by which cells remove a potentially toxic retroviral RNA and avoid damage.

## 2. Materials and Methods

### 2.1. Mice and Ethics

Mice were bred and maintained in the animal facilities at the Walter and Eliza Hall Institute of Medical Research (WEHI) according to national and institutional guidelines for animal care. All experimental procedures were approved by the WEHI Animal Ethics Committee (project number 2014.019, 5 September 2014).

### 2.2. Cell Culture

To obtain bone-marrow-derived DCs (BMDCs), femurs from C57Bl/6 mice were flushed with Dulbecco’s modified Eagle’s medium (DMEM) (Themofisher Scientific Australia Pty. Ltd., Scoresby, VIC, Australia). The resulting bone marrow cells were resuspended, counted, plated at a density of 2 million cells per 10 cm petri dish, and then cultured for 7 days at 37 °C with 5% CO_2_ in DMEM supplemented with 10% fetal calf serum (FCS) (Sigma-Aldrich, Castle Hill, NSW, Australia) and 10% X-63 (GM-CSF) supernatant to allow the cells to differentiate into DCs. Immortalised cell lines, including a mouse dendritic cell line (DC2.4), a mouse thymoma cell line (EL4), a mouse B cell lymphoma line (WEHI-231), a mouse fibroblast line (NIH/3T3), the human embryonic kidney cell line (HEK293T), and a human neuroblastoma cell line (SH-SY5Y) were cultured in DMEM supplemented with L-glutamine (2 mM), streptomycin (100 μg/mL), and penicillin (100 U/mL), nonessential amino acid (100 μM), 2-mercaptoethanol (2-ME, 50 μM)(supplements purchased from Sigma-Aldrich) and 10% FCS and cultured at 37 °C and 5% CO_2_.

### 2.3. SEV Isolation

SEV-free medium was prepared by: pelleting any SEVs in DMEM/20% FCS via ultracentrifugation at 100,000 *g* using an SW-28 rotor (Beckman Coulter, Mount Waverley, VIC, Australia) for 16 h at 4 °C; removing the supernatant; diluting this 1:1 in DMEM; and filtering the resultant solution with a 0.2 μm filter. To isolate SEVs, cells were first plated and grown in SEV-free medium for 48 h. Supernatant was then collected, centrifuged at 1500 rpm to remove cellular debris, and then filtered with a 0.2 μm filter to remove microparticles. SEVs were pelleted by ultracentrifugation at 100,000× *g* using an SW-28 rotor for one hour at 4 °C. Then, EV pellets were resuspended in 1 × sterile PBS (Sigma-Aldrich) and spun down at 100,000× *g* for an hour. Finally washed pellets were diluted with either 50 μL of 1 × PBS for Western blot or 250 μL of 1 × PBS for RNA isolation. For experiments involving RNase treatment of SEVs, 250 μL SEV solution was treated with 5 mg/mL RNAse A (Sigma-Aldrich Corp., St. Louis, MO, USA) and incubated at 37 °C for 5 min, prior to RNA isolation. SEV pellets were stored at −80 °C until use.

### 2.4. SEV Size Assessment

SEV size distribution was assessed via tunable resistive pulse sensing using a qNano instrument (Izon Sciences, Christchurch, NZ) according to a previously published protocol [14]. The qNano instrument was initially calibrated using polystyrene beads (CPC100, CP100 and CPC200 depending on which nanopore size was used). Once a stable current was established, 40 µL SEV samples in PBS were filtered and introduced to the sample fluid cell. NP800, NP400 and NP150 nanopore filters were used. Each nanopore was stretched to 44.75 mm and 0.6 kPa pressure was applied using the variable pressure module (VPM). SEV samples were passed through 0.8 μm, 0.45 μm or 0.22 μm filters depending on the nanopore size being used. Data were analysed using Izon Control Suite software (Izon Sciences).

### 2.5. RNA Isolation

Total RNA was extracted from cells using Tri-Reagent (Sigma-Aldrich) as per the manufacturers’ instructions. SEV RNA was extracted using Trizol LS (Themofisher Scientific Australia Pty. Ltd., Scoresby, VIC, Australia) then purified further using Qiagen RNA Mini kit (Qiagen Pty. Ltd., Chadstone, VIC, Australia). RNA yield was quantified using NanoDrop (Themofisher Scientific Australia Pty. Ltd.) and RNA quality was assessed using either the TapeStation System 2200 or the Bioanalyser (Agilent Technologies, Mulgrave, VIC, Australia). To remove contaminating DNA, 1 μg RNA was treated with 1 μL (2 U) of DNAse I (Themofisher Scientific Australia Pty. Ltd.) in a 20 μL reaction incubated at 37 °C for 30 min and 1 μL DNAse inactivation reagent added.

### 2.6. RNA-Seq

RNA libraries were prepared from 200 ng of total RNA using a TruSeq RNA library preparation kit v.2 (Illumina, Melbourne, VIC, Australia) and RNA-seq was performed on an Illumina NextSeq550 sequencing platform (Illumina), generating 80 base pair (bp) paired-end reads. The number of reads overlapping each mouse Entrez gene were summarized using featureCounts of the R subread package (https://www.rstudio.com/products/rpackages/, accessed on 6 July 2021) [15]. Entrez genes were identified using NCBI RefSeq annotation. Differential expression analyses were undertaken for the cell and SEV samples using the edgeR [16] and limma [17] software packages (https://www.rstudio.com/products/rpackages/, accessed on 6 July 2021). For the cell samples, any gene that did not achieve an average count per million mapped reads (CPM) greater than 0.15 in at least 50% of samples was deemed to not be expressed and subsequently filtered out. For the SEV samples, genes were filtered out if they failed to achieve a CPM greater than 0.05 in at least three samples. Compositional differences between cell and SEV libraries were normalised using the trimmed mean of log expression ratios (TMM) method [18], and counts were then transformed to log_2_CPM with associated prevision weights using mean-variance modelling at the observational level (voom, http://www.bioconductor.org, accessed on 6 July 2021) [19]. Differential expression between the cell and SEV samples was assessed using linear models and robust empirical Bayes moderated t-statistics [20]. p-values were adjusted to control the false discovery rate (FDR) below 5% using the Benjamini and Hochberg method.

### 2.7. QRT-PCR

One microgram of DNAse-treated RNA was reverse transcribed using Superscript II reverse transcriptase (Themofisher Scientific Australia Pty. Ltd.) and oligo(dT)_15_ primers (Promega, Alexandria, NSW, Australia) according to the manufacturers’ instructions. Real-time PCR was performed using AccuPower Greenstar qPCR Mastermix (Bioneer Pacific, Kew East, VIC, Australia) and run on a LightCycler 480 (Roche, Sydney, NSW, Australia). The following primers were used: VL30-qRTPCR (Fwd: TCCCTATGCTGACCACTTCC; Rev: TTTTTCCCTAGGCTCCAGGT); Motif –BamH1-EcorI-XhoI (Fwd: aaaaggatccTCAGTTTTGCGGATGCTCAG; Rev: ttttgaattcttttctcgagGAAGAATCAGATGGCCTCTCTAAG); VL30-BamHI-EcorI (Fwd: AAAAGGATCCAAAAGAATTCGGGTTCGAGTCCCACCTCGTGCAGAGGGTCTC; Rev: AAAAGAATTCTCTAACCCACGATCTCGCAA); mouse β-actin (Fwd: CTGTCCCTGTATGCCTCTG; Rev: ATGTCACGCACGATTTCC); human β-actin (Fwd: CACAGCTGAGGGAAATC; Rev: CACTGTGTTGGCATAGAGG); Ifit1 (Fwd: ATGGGAGAGAATGCTGATGG; Rev: AGGAACTGGACCTGCTCTGA); Irf7 (Fwd: CCAGTTGATCCGCATAAGGT; Rev: AGCATTGCTGAGGCTCACTT); MDA5 (Fwd: TCACTGATCTGCCCTCTCCT; Rev: CCTTCTCGAAGCAAGTGTCC); RIG-I (Fwd: AAAGACGGTTCACCGCATAC; Rev: TCTTGCACTTTCCACACAGC)

### 2.8. Transmission Electron Microscopy (TEM)

TEM analysis of SEV samples was performed as previously described [21]. Briefly, 50 μL SEV pellet was dissolved in 4.84 mM EDTA/DPBS and fixed with 1% glutaraldehyde overnight at 4 °C. Aliquots were then absorbed onto glow-discharged 200-mesh formvar with carbon coating Cu grids (ProSciTech, Thuringowa Central, QLD, Australia) the next day. Grids were washed twice with MilliQ water and negatively stained with 2% uranyl acetate. Images were captured using a Tecnai G2 F30 transmission electron microscope (FEI, Hillsboro, OR, USA), operating at 300 kV (Bio21 Institute, Melbourne).

### 2.9. VL30 Cloning

Primers to amplify full length and motif only-containing VL30 inserts were designed and each fragment was amplified by PCR. Primers were designed to include BamHI and EcoRI restriction sites at the ends of each construct and, following restriction enzyme digestion, purified VL30 full length and motif-only containing fragments were ligated into an empty lentiviral plasmid (pfTREtight-rtTAadvanced_puro, a gift from T. Okamoto and D. Huang) using T4-DNA ligase (Promega) following the manufacturer’s instructions. Plasmids were then transformed into DH5 alpha competent cells (Sigma-Aldrich). Virus was produced in HEK-293T cells and finally transduced into SH-SY5Y cells and stable cell lines selected using puromycin (Sigma-Aldrich). For doxycycline treatment, VL30-transduced SH-SY5Ys were plated in T175 cm flasks; once cells reached 80% confluency, they were treated with 1 μg/mL doxycycline (Sigma-Aldrich) for 48 h to induce expression of motif only and full length VL30.

### 2.10. Motif Discovery Search and RNA Secondary Structure Prediction

Full length VL30 sequences were uploaded to MEME Suite 5.0.2 (https://meme-suite.org/meme/, accessed on 6 July 2021) [22], and motifs occurring at a minimum of two sites with any number of repeats were searched across both positive and negative strands. RNA secondary structure predictions were performed via Mfold (http://mfold.rna.albany.edu/?q=mfold/download-mfold, accessed on 6 July 2021) [23] using default parameters.

### 2.11. Western Blots

SEV pellets were suspended in a minimum volume of PBS and mixed with lysis buffer containing the following chemicals purchased from Sigma-Aldrich (Themofisher Scientific Australia Pty. Ltd.): 150 mM NaCl, 50 mM Tris at pH 7.4, 1% (*v*/*v*) Triton X-100, 0.5% (*w*/*v*) sodium deoxycholate) supplemented with protease inhibitor cocktail tablet (Roche, Sydney, NSW, Australia). Cells were permeabilised in the following chemicals: 0.025% digitonin with 20 mM HEPES-NaOH pH 7.5 (Sigma-Aldrich), 100 nM sucrose (Sigma-Aldrich), 2.5 mM MgCl_2_ (Sigma-Aldrich), 100 mM KCl (Sigma-Aldrich), supplemented with protease inhibitors (Roche, Sydney, NSW, Australia) for 10 min and sonicated for 2 min. Homogenates were then spun down at 13,000 *g* at 4 °C for 15 min. Total protein in the resultant supernatant was quantified using Pierce BCA Protein Assay kit (Thermofisher Scientific Australia Pty. Ltd). Forty to fifty micrograms of protein per lane were loaded onto 4–12% NuPAGE precast (Thermofisher Scientific Australia Pty. Ltd) gels, separated by SDS-PAGE, and transferred to polyvinylidene fluoride membranes (Merck Millipore, Bayswater, VIC, Australia) at 100 volts for an hour. Membranes were then blocked with 5% commercial BSA (Sigma-Aldrich) in 1 × TBS, 0.1% Tween 20 (Sigma-Aldrich) for 30 min then probed with the following antibodies overnight at 1:1000 dilution: FLOT1 (Mouse monoclonal, Cat. No. 610821, BD Biosciences, Mulgrave, VIC, Australia), TSG-101 (Rabbit polyclonal, Cat. No. T5701, Sigma-Aldrich), Transferrin Receptor (Rabbit monoclonal, Cat. No. 13208, Cell Signaling Technologies, Danvers, MA, USA), GM130 (Mouse monoclonal, Clone 4A3, Cat. No. 32160702, Sigma-Aldrich), Bcl-2 (Mouse Monoclonal, Cat. No. 610539, BD Biosciences), Calnexin (Rabbit polyclonal, Cat. No. ab22595 Abcam, Cambridge, MA, USA) and VDAC-1 (Mouse monoclonal, Cat. No. ab14734 Abcam). Blots were then washed three times for 10 min, probed with antirabbit secondary-HRP antibody for an hour, washed as before then treated with Luminata Forte Western HRP substrate (Millipore), and visualised using the ChemiDoc MP system (Bio-Rad, Gladesville, NSW, Australia).

### 2.12. Statistics

Statistical analyses to compare RNA levels in qRT-PCR experiments were performed using unpaired, two-tailed Student’s *t*-tests using Prism 7.0 software (GraphPad Software, San Diego, CA, USA). All histograms show mean values, with error bars indicating standard error of the mean (SEM) unless otherwise stated.

## 3. Results

### 3.1. The VL30 lncRNA Is Enriched in DC SEVs

**Figure 1:**
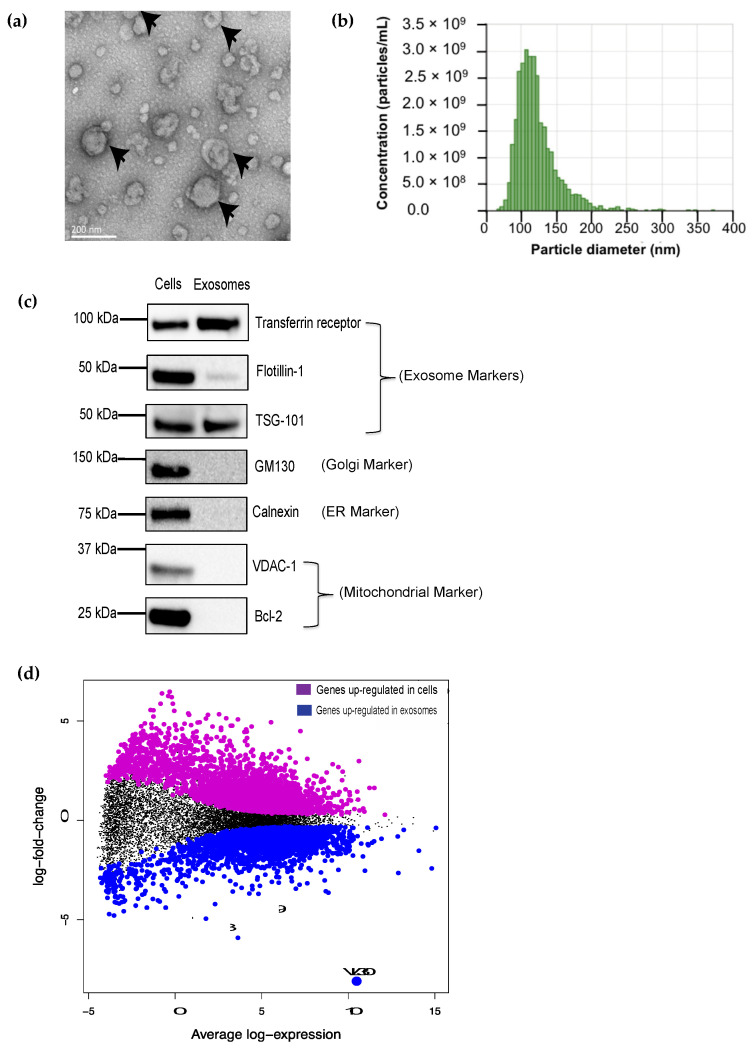
The VL30 RNA is enriched in DC SEVs. (**a**) DC SEVs (black arrows) display a typical appearance by transmission EM; scale bar = 200 μm. (**b**) Size assessment of SEVs by qNano. Most vesicles fall within the size range expected for SEVs (60–140 nm). (**c**) Western blot analysis of classical SEV markers–transferrin receptor, flotillin-I and TSG-101–from cell and SEV DC lysates. GM130 (Golgi), Calnexin (ER), VDAC-1 and Bcl-2 (mitochondria) proteins were not detected in the DC SEVs indicating a lack of contamination by cell debris. Each lane loaded with 50 μg protein. (**d**) MA plot showing the average log expression of each gene (horizontal axis) plotted against the log-fold change in gene expression between cells and SEVs (vertical axis), based on RNA sequencing of DCs and their SEVs (n = 6 each). Each dot in the graph represents one RNA. Transcripts that were differentially expressed between cells and SEVs were identified (adjusted *p*-value < 0.05; see Methods) and are shown either in magenta (upregulated in cells) or blue (upregulated in SEVs). The RNA that showed the greatest enrichment in SEVs (~200-fold) was VL30 (big blue dot).

To first isolate SEVs, we performed ultrafiltration and differential ultracentrifugation of culture supernatant from mouse BMDCs, which are a rich source of SEVs [24]. The resultant extracellular vesicles appeared round and membrane-bound via electron microscopy (Figure 1a) and their size ranged from 50–200 nanometers (mean: ~120 nm) (Figure 1b), in keeping with the expected appearance and size of SEVs [25]. Western blotting was also consistent with these vesicles being SEVs, as indicated by the presence of classical SEV markers such as transferrin receptor, flotillin-1 and TSG101 and the absence of markers for the Golgi (GM130), endoplasmic reticulum (Calnexin) and mitochondria (VDAC-1/Bcl-2) (Figure 1c).

To identify RNAs enriched in SEVs, we next isolated total RNA from these SEVs and their parental DCs, prepared cDNA libraries, and performed RNA-Seq. A total of 7968 genes were identified as differentially expressed when comparing the cell and SEV transcriptomes (Figure 1d). Of the 3496 genes upregulated in SEVs, >90% showed only a mild increase in relative abundance (log_2_ fold change <2). Even among the top 20 RNAs that were most enriched in SEVs, there was one RNA known as VL30 (gene symbol: A130040M12Rik) that stood out based not only on its strong enrichment (~200-fold) but also its overall abundance (Figure 1d, Supplementary Table S1).

### 3.2. The VL30 lncRNA Is Enriched in SEVs from Multiple Cell Types

**Figure 2:**
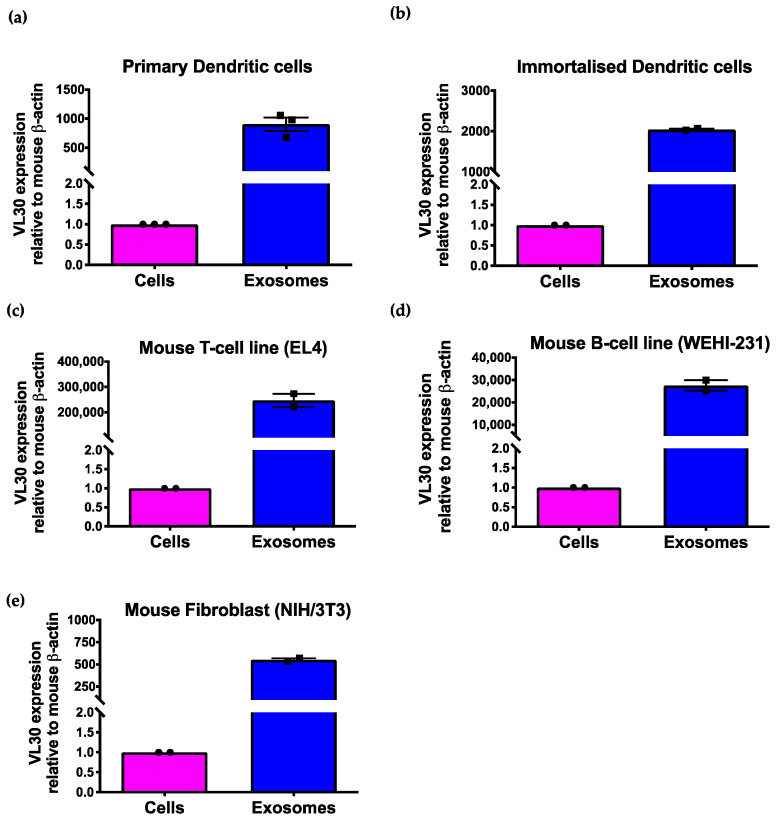
VL30 RNA is enriched in SEVs from multiple cell types. Relative abundance of VL30 RNA normalized to β-actin in cells and SEVs for (**a**) primary dendritic cells, (**b**) immortalized dendritic cells, (**c**) T cells, (**d**) B cells and (**e**) fibroblasts. Data shown are averages of three independent experiments. Error bars represent SEM.

VL30 is a long noncoding RNA that is derived from a mouse-specific endogenous retrovirus and functions as a transcriptional regulator in steroidogenesis and oncogenesis [26,27]. To explore whether VL30 is also enriched in SEVs from other cell types, we next cultured a variety of different mouse cell lines, including immortalized DCs (DC2.4), T cells (EL4), B cells (WEHI-231) and fibroblasts (NIH/3T3), and isolated SEVs from each of these lines. VL30 abundance in SEVs was then compared to that of each parental cell line by qRT-PCR, using β-actin for normalization purposes because of its high abundance and consistent average expression in both cells (~8400 CPM) and SEVs (~6300 CPM) as observed in our original RNA-Seq data. Consistent with our data from primary DCs, VL30 was significantly enriched in SEVs from each of the tested cell lines, with an abundance in SEVs ranging from 500- to 300,000-fold higher than the parental cells (Figure 2).

### 3.3. VL30 RNA Isoforms Enriched in SEVs Contain a Repeated Sequence Motif

**Figure 3:**
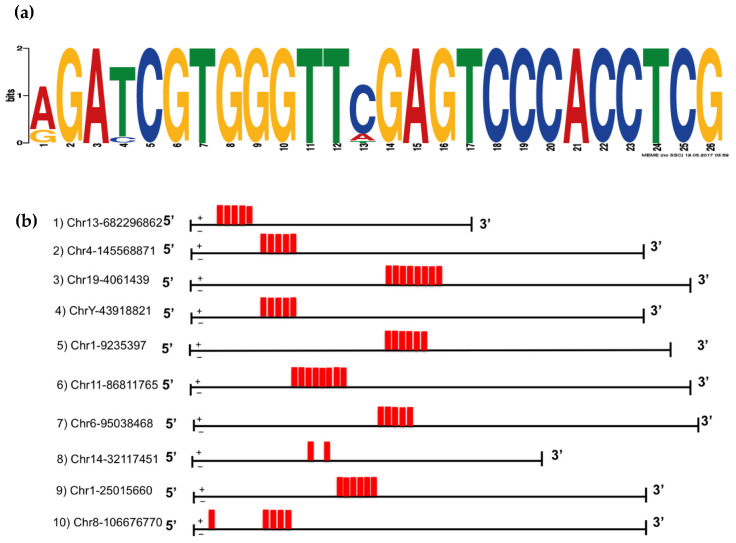
SEV-enriched VL30 isoforms contains a tandemly-repeated motif. (**a**) Using MEME, a 26 nucleotide motif was identified within the ten VL30 isoforms that showed the greatest SEV enrichment. (**b**) The location of this motif (red box) is shown within each of the ten VL30 isoforms that showed the greatest SEV enrichment. The chromosomal location of the 5‘-end for each isoform is shown (mm10).

Consistent with its retrotransposon origin, the VL30 gene has multiple copies (>400) throughout the mouse genome [28]. Over time, these sequences have diverged considerably. To understand the possible sequence requirements for the VL30 RNA to be efficiently incorporated into SEVs, multiple VL30 isoforms from across the genome were evaluated based on the number of counts present in the SEV and cell-based libraries within our original RNA-Seq data. Specifically, the ten most SEV-enriched VL30 isoforms were selected and their sequences assessed using the Multiple Expectation maximizations for Motif Elicitation (MEME) tool, which enables motif discovery among related sequences. This revealed a 26 nucleotide motif (Figure 3a) that exists in tandem repeats within SEV-enriched VL30 isoforms (Figure 3b) but is absent from the ten VL30 isoforms that showed the least SEV enrichment.

### 3.4. A VL30 Sequence Containing the Repeated Motif Alone Is Efficiently Incorporated into SEVs

**Figure 4:**
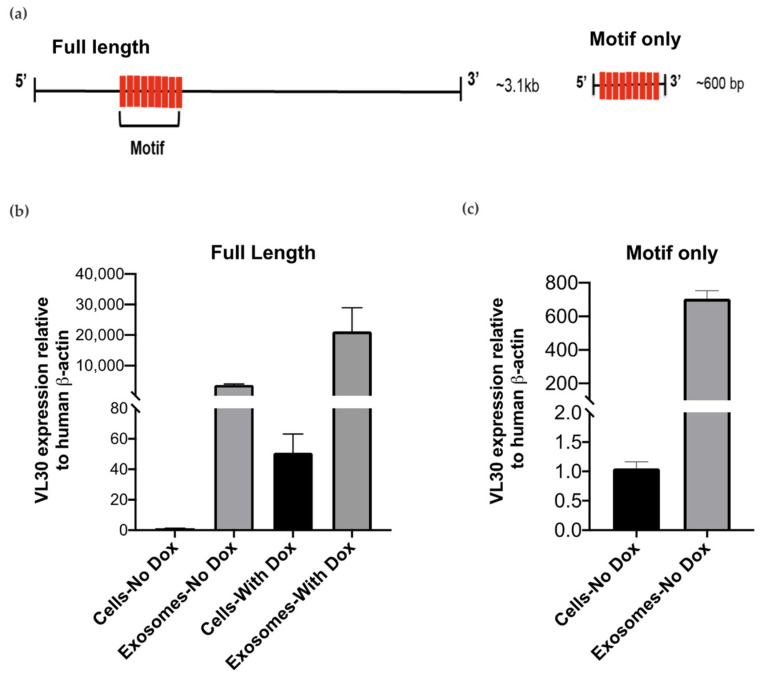
Full-length VL30 lncRNA and motif sequence were incorporated in SEVs of human neuronal cell line (SH-SY5Y). (**a**) Schematic representation of full length and motif-only VL30 constructs derived from the C730003K16 Riken clone. Both constructs contain nine tandem copies of the 26-bp motif sequence (red bar). (**b**) SH-SY5Y cells were transduced with a full length VL30 construct under the control of a doxycycline-inducible promoter. Abundance of VL30 RNA in both cells and SEVs ± doxycycline treatment was measured by qRT-PCR, and normalised to β–actin. Data shown are representative of two independent experiments. Error bars represent SEM. (**c**) SH-SY5Y cells were transduced with the motif-only VL30 construct under the control of a doxycycline-inducible promoter. Abundance of VL30 RNA in both cells and SEVs ± doxycycline treatment was measured by qRT-PCR, and normalised to β–actin. Data shown are representative of two independent experiments. Error bars represent SEM. Given widespread cell death following addition of doxycycline, only data obtained in the absence of doxycycline are shown for the cells transduced with motif alone.

To test whether this repetitive motif was important for packaging into SEVs, a full-length cDNA clone of VL30 (C730003K16) containing nine tandem copies of the motif was obtained, and a truncated, “motif-only” construct representing ~20% of the full-length sequence and containing the repetitive motif alone was generated (Figure 4a). The full length and motif-only constructs were then separately cloned into a doxycycline-inducible lentiviral vector and transduced into the SH-SY5Y human neuroblastoma cells, which are a rich source of SEVs and lack VL30 expression.

To first test whether expression of the full length construct was associated with enrichment of VL30 in SH-SY5Y SEVs, SEVs from SH-SY5Y cells were isolated and the relative abundance of VL30 RNA in cells and SEVs was compared by qRT-PCR (Figure 4b). In the absence of doxycycline, VL30 was readily detected within cells (presumably due to ‘leakiness’ of the doxycycline-inducible promoter as commonly occurs) and enriched >5000-fold in SEVs. A similar SEV enrichment (~20,000 fold) was observed following doxycycline treatment, which as expected increased overall VL30 levels within the cells themselves. Together, these results suggested that the full length VL30 construct could be successfully overexpressed and efficiently packaged into SEVs.

We next turned our attention to the motif-only construct. Here, we observed that, similar to the full length construct, VL30 containing the repeated motif alone was detected in cells even in the absence of doxycycline and highly enriched in SEVs (~600-fold) (Figure 4c), consistent with the tandem repeat of the motif being itself sufficient to promote SEVs loading. However, when doxycycline was added to induce overexpression of the motif-only construct, widespread cell death was unexpectedly observed, which was not the case for the full length construct (Supplementary Figure S1).

**Figure 5:**
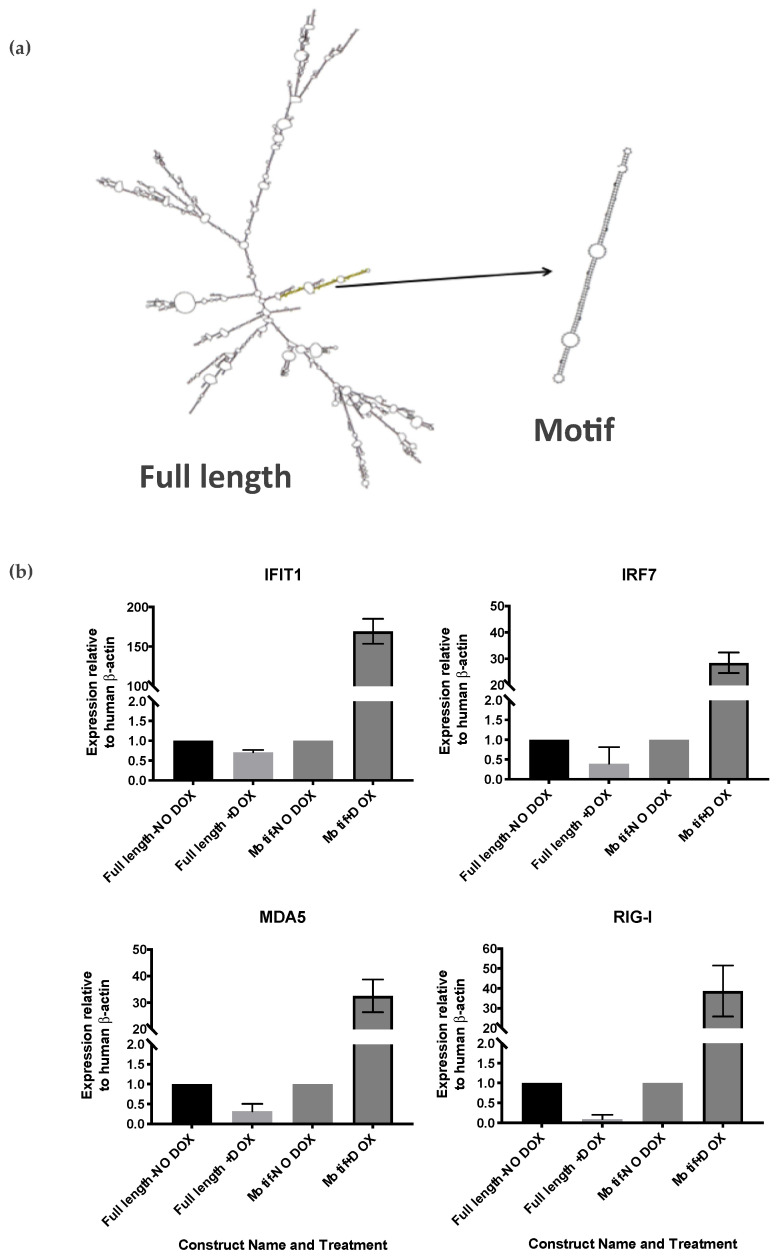
VL30 contains a long dsRNA motif whose overexpression induces interferon-stimulated gene expression. (**a**) Secondary structures of full length VL30 (left) and Motif only (right) generated by MFold. The VL30 motif forms a dsRNA-like structure. (**b**) Relative expression of IFIT1, IRF7, MDA5 and RIG-I in SH-SY5Y cells transduced with full length VL30 and motif only constructs in the presence or absence of doxycycline. Data represent technical triplicates and error bars represent SEM.

To investigate this further, we examined the likely secondary structure of our motif-only VL30 RNA using Mfold, and found that this RNA is strongly predicted to form a long dsRNA hairpin (Figure 5a). Given that dsRNA is a potent pathogen associated molecular pattern that induces a type I IFN response and cell death, we performed qRT-PCR for IFN-β as well as several common interferon-stimulated genes (ISGs), including IFIT1, IRF7, MDA5 and RIG-I 48 h after doxycycline treatment. While we could not reliably detect any IFN-β, which is often produced at very low levels and is notoriously difficult to detect, each of the ISGs showed strong up-regulation upon doxycycline induction of the motif-only VL30 RNA but not the full-length construct (Figure 5b).

## 4. Discussion

Since the identification of RNA in SEVs [29] and the discovery that SEVs can facilitate the transfer of RNAs into recipient cells [30,31], there has been growing interest in the use of SEVs as a ‘natural delivery system’ for therapeutic RNAs. To date, however, there have been very few studies examining the loading requirements for RNAs to be selectively packaged into SEVs. The original motivation for this study was therefore to better understand why certain RNAs are loaded into SEVs. Our subsequent identification of VL30 as an RNA that is highly enriched in SEVs (up to several thousand-fold) provided an opportunity to examine the features of this RNA that promote SEV loading. In this regard, we observed that highly enriched isoforms of VL30 contained multiple copies of a 26 nucleotide motif, and we then demonstrated that a small fragment of the VL30 RNA containing tandem repeats of this motif was sufficient for strong SEV enrichment.

In theory, our identification of an RNA sequence that promotes SEV loading could assist efforts to selective package therapeutic RNAs into SEVs. However, overexpression of the repeated motif led to induction of a strong type I IFN response, consistent with its dsRNA structure. If the motif were added to the sequence of a therapeutic RNA as a means of promoting SEV loading, it would therefore be important to avoid this innate immune response; otherwise the health of the parental cells and their SEV production would be compromised, as we observed. In this regard, it is interesting to note that overexpression of the full length VL30 sequence—despite it containing the same repeated motif in its entirety—did not induce a type I IFN response. Why this should be the case is unclear, but one possibility is that the tertiary folding of the full length VL30 RNA either prevents the repeated motif’s dsRNA structure from forming in the first place or else hides it internally so as to prevent recognition by innate immune receptors such as MDA5, RIG-I and TLR3 that recognize dsRNA. Whether adding a therapeutic RNA to the repeated motif enables selective loading of the RNA into SEVs and/or helps to similarly avoid a type I IFN response remains to be seen.

Our observation that VL30 RNA was enriched in SEVs from a wide variety of cell types as well as from different species (mouse and human) suggests that the features of VL30 that promote its selective packaging into SEVs utilize a cellular mechanism that is widespread and evolutionarily conserved. What that mechanism might be is something for future study, but we can speculate as to the features of the VL30 RNA that promote its SEV loading. Firstly, by conducting a BLAST search of the 26 nucleotide motif against the NCBI nucleotide database (and excluding inevitable hits to VL30 itself within the mouse genome), we found that the motif matched the viral packaging signal (Psi) that is contained within various retroviral/retrotransposon-based vectors. This signal, originally derived from the VL30 retrotransposon, is believed to efficiently direct the packaging of recombinant RNAs, such as those from the reporter gene lacZ, into virions [32,33]. At first glance, the observation that the same signal is involved in virion and SEV packaging might seem surprising, but it would be entirely consistent with the “Trojan exosome hypothesis” which proposes that retroviruses have come to exploit our bodies’ exosome biogenesis pathways for the purposes of producing retroviral particles [34,35]. Secondly, the predicted dsRNA structure of the repeated motif is in keeping with an earlier report from Botagov and colleagues that RNAs enriched in SEVs contain dsRNA hairpin structures [11]. It is also consistent with previous observations that human immunodeficiency virus (HIV) transactivating response (TAR) RNA, which also contains a dsRNA structure [36], is highly enriched in SEVs [37]. Taken together with our own findings, these previous studies therefore suggest the dsRNA might itself be a signal for selective SEV loading.

If dsRNA is indeed a signal for selective SEV loading, a question that arises is what purpose this might serve. During viral infection, the extracellular transfer of viral dsRNA from infected cells has been proposed as a means of activating the innate immune response within noninfected bystander cells, thus augmenting antiviral immunity in the face of the various immunosuppressive mechanisms that viruses employ within infected cells [38]. Consistent with this, SEVs from HIV-infected cells contain TAR RNA that activates TLR-3 and stimulates proinflammatory cytokine production [39]. Similarly, cells infected with hepatitis virus C produce SEVs that transfer viral RNAs to recipient cells and trigger the production of type I IFN [30], although in this case it was unclear whether the immunostimulatory viral RNAs were double-stranded. Nevertheless, the selective loading of dsRNA into SEVs represent a feasible strategy for infected cells to augment antiviral immunity. But what about if there was no viral infection? Cells produce an abundance of endogenous dsRNAs, and there are a variety of mechanisms to ensure that these do not cause unwanted autoinflammatory responses [40]. In this regard, it is tempting to speculate that the selective packaging of dsRNA into SEVs might provide an additional mechanism for cells to remove this material and therefore avoid autoinflammation. Such a role would be in keeping with the abundance of SEVs that are continually excreted in urine and other bodily fluids and would hark back the original notion of SEVs as a cellular waste bin [1].

Our study is not without several important limitations. Firstly, we are aware that it would have been helpful to further define the minimum VL30 sequence that facilitates SEV loading. In this regard, it is notable that previous work with the VL30 Psi sequence in relation to retroviral packaging suggests that a Psi sub-sequence of as little as 61 nucleotides is sufficient for promoting RNA encapsidation (albeit with somewhat reduced efficiency) [32]. It would therefore be interesting to determine if the same minimal sequence can facilitate SEV loading in the future. At the same time, removing the motif sequence from VL30 and testing whether VL30 is still enriched within SEVs would provide further confirmation that the motif is required for SEV loading. Secondly, another limitation of the present study is that we did not test whether our putative VL30 SEV loading sequence was able to facilitate the loading of a reporter RNA. This would have allowed us to provide direct proof-of-concept that therapeutic RNAs can be more efficiently packaged into SEVs through the addition of a VL30 sequence. Finally, looking ahead, it would be important to identify the cellular components that facilitate VL30 RNA loading into SEVs. In this regard, RNA pull-down assays using tagged VL30 RNA to isolate and identify the proteins that interact with VL30 would be informative and ultimately facilitate a proper understanding of why and how VL30 is so efficiently loaded into SEVs.

## 5. Conclusions

In this study, we observed that the VL30 RNA is highly enriched in SEVs from multiple cell types, and identified a tandemly-repeated motif that appears to help promote the selective loading of VL30 into SEVs. Further study into whether this repetitive motif can help promote loading of therapeutic RNAs into SEVs is warranted.

## Data Availability

The data presented in this study are available on request from the corresponding author.

## Supplementary Materials

The following are available online at https://www.mdpi.com/article/10.3390/biomedicines9091136/s1, Figure S1: Overexpression of the motif-only construct in SH-SY5Y cells results in widespread cell death, Table S1: Top 20 RNAs most enriched in cells or exosomes.

## References

[1] Johnstone R.M. (1992). Maturation of reticulocytes: Formation of exosomes as a mechanism for shedding membrane proteins. Biochem. Cell Biol..

[2] Patil S.M., Sawant S.S., Kunda N.K. (2020). Exosomes as drug delivery systems: A brief overview and progress update. Eur. J. Pharm. Biopharm..

[3] Bunggulawa E.J., Wang W., Yin T., Wang N., Durkan C., Wang Y., Wang G. (2018). Recent advancements in the use of exosomes as drug delivery systems. J. Nanobiotechnol..

[4] Aslan C., Kiaie S.H., Zolbanin N.M., Lotfinejad P., Ramezani R., Kashanchi F., Jafari R. (2021). Exosomes for mRNA delivery: A novel biotherapeutic strategy with hurdles and hope. BMC Biotechnol..

[5] O’Brien K., Breyne K., Ughetto S., Laurent L.C., Breakefield X.O. (2020). RNA delivery by extracellular vesicles in mammalian cells and its applications. Nat. Rev. Mol. Cell Biol..

[6] Koppers-Lalic D., Hackenberg M., Bijnsdorp I.V., van Eijndhoven M.A., Sadek P., Sie D., Zini N., Middeldorp J., Ylstra B., de Menezes R.X. (2014). Nontemplated nucleotide additions distinguish the small RNA composition in cells from exosomes. Cell Rep..

[7] Villarroya-Beltri C., Baixauli F., Gutiérrez-Vázquez C., Sánchez-Madrid F., Mittelbrunn M. (2014). Sorting it out: Regulation of exosome loading. Seminars in Cancer Biology.

[8] Villarroya-Beltri C., Gutierrez-Vazquez C., Sanchez-Cabo F., Pérez-Hernández D., Vázquez J., Martin-Cofreces N., Martinez-Herrera D.J., Pascual-Montano A., Mittelbrunn M., Sánchez-Madrid F. (2013). Sumoylated hnRNPA2B1 controls the sorting of miRNAs into exosomes through binding to specific motifs. Nat. Commun..

[9] Groot M., Lee H. (2020). Sorting mechanisms for MicroRNAs into extracellular vesicles and their associated diseases. Cells.

[10] Bolukbasi M.F., Mizrak A., Ozdener G.B., Madlener S., Ströbel T., Erkan E.P., Fan J.B., Breakefield X.O., Saydam O. (2012). miR-1289 and “Zipcode”-like Sequence Enrich mRNAs in Microvesicles. Mol. Ther.-Nucleic Acids.

[11] Batagov A.O., Kuznetsov V.A., Kurochkin I.V. (2011). Identification of nucleotide patterns enriched in secreted RNAs as putative cis-acting elements targeting them to exosome nano-vesicles. BMC Genom..

[12] Kossinova O.A., Gopanenko A.V., Tamkovich S., Krasheninina O., Tupikin A.E., Kiseleva E., Yanshina D.D., Malygin A., Ven’Yaminova A.G., Kabilov M. (2017). Cytosolic YB-1 and NSUN2 are the only proteins recognizing specific motifs present in mRNAs enriched in exosomes. Biochim. Biophys. Acta (BBA)-Proteins Proteom..

[13] Pitt J.M., André F., Amigorena S., Soria J.-C., Eggermont A., Kroemer G., Zitvogel L. (2016). Dendritic cell–derived exosomes for cancer therapy. J. Clin. Investig..

[14] Maas S.L.N., De Vrij J., Broekman M.L.D. (2014). Quantification and size-Profiling of extracellular vesicles using tunable resistive pulse sensing. J. Vis. Exp..

[15] Liao Y., Smyth G.K., Shi W. (2014). Feature counts: An efficient general purpose program for assigning sequence reads to genomic features. Bioinformatics.

[16] Robinson M.D., McCarthy D.J., Smyth G.K. (2009). edgeR: A Bioconductor package for differential expression analysis of digital gene expression data. Bioinformatics.

[17] Ritchie M.E., Phipson B., Wu D., Hu Y., Law C.W., Shi W., Smyth G.K. (2015). Limma powers differential expression analyses for RNA-sequencing and microarray studies. Nucleic Acids Res..

[18] Robinson M.D., Oshlack A. (2010). A scaling normalization method for differential expression analysis of RNA-seq data. Genome Biol..

[19] Law C.W., Chen Y., Shi W., Smyth G.K. (2014). voom: Precision weights unlock linear model analysis tools for RNA-seq read counts. Genome Biol..

[20] Phipson B., Lee S., Majewski I., Alexander W.S., Smyth G.K. (2016). Robust hyperparameter estimation protects against hypervariable genes and improves power to detect differential expression. Ann. Appl. Stat..

[21] Bellingham S.A., Coleman B.M., Hill A.F. (2012). Small RNA deep sequencing reveals a distinct miRNA signature released in exosomes from prion-infected neuronal cells. Nucleic Acids Res..

[22] Bailey T.L., Elkan C. (1994). Fitting a mixture model by expectation maximization to discover motifs in bipolymers. Proc. Int. Conf. Intell. Syst. Mol. Biol..

[23] Zuker M. (2003). Mfold web server for nucleic acid folding and hybridization prediction. Nucleic Acids Res..

[24] Théry C., Boussac M., Véron P., Ricciardi-Castagnoli P., Raposo G., Garin J., Amigorena S. (2001). Proteomic analysis of dendritic cell-derived exosomes: A secreted subcellular compartment distinct from apoptotic vesicles. J. Immunol..

[25] Théry C., Witwer K.W., Aikawa E., Alcaraz M.J., Anderson J.D., Andriantsitohaina R., Antoniou A., Arab T., Archer F., Atkin-Smith G.K. (2018). Minimal information for studies of extracellular vesicles 2018 (MISEV2018): A position statement of the International Society for Extracellular Vesicles and update of the MISEV2014 guidelines. J. Extracell Vesicles.

[26] Song X., Sui A., Garen A. (2004). Binding of mouse VL30 retrotransposon RNA to PSF protein induces genes repressed by PSF: Effects on steroidogenesis and oncogenesis. Proc. Natl. Acad. Sci. USA.

[27] Song X., Wang B., Bromberg M., Hu Z., Konigsberg W., Garen A. (2002). Retroviral-mediated transmission of a mouse VL30 RNA to human melanoma cells promotes metastasis in an immunodeficient mouse model. Proc. Natl. Acad. Sci. USA.

[28] Markopoulos G., Noutsopoulos D., Mantziou S., Gerogiannis D., Thrasyvoulou S., Vartholomatos G., Kolettas E., Tzavaras T. (2016). Genomic analysis of mouse VL30 retrotransposons. Mob. DNA.

[29] Valadi H., Ekström K., Bossios A., Sjöstrand M., Lee J.J., Lötvall J.O. (2007). Exosome-mediated transfer of mRNAs and microRNAs is a novel mechanism of genetic exchange between cells. Nat. Cell Biol..

[30] Dreux M., Garaigorta U., Boyd B., Décembre E., Chung J., Whitten-Bauer C., Wieland S., Chisari F.V. (2012). Short-Range exosomal transfer of viral rna from infected cells to plasmacytoid dendritic cells triggers innate immunity. Cell Host Microbe.

[31] Skog J., Würdinger T., Van Rijn S., Meijer D.H., Gainche L., Curry W.T., Carter B.S., Krichevsky A.M., Breakefield X.O. (2008). Glioblastoma microvesicles transport RNA and proteins that promote tumour growth and provide diagnostic biomarkers. Nature.

[32] Darlix J.-L., Torrent C. (1998). Retroviral Vectors Comprising a VL30-Derived psi Region. U.S. Patent.

[33] Torrent C., Gabus C., Darlix J.L. (1994). A small and efficient dimerization/packaging signal of rat VL30 RNA and its use in murine leukemia virus-VL30-derived vectors for gene transfer. J. Virol..

[34] Gould S.J., Booth A.M., Hildreth J.E.K. (2003). The Trojan exosome hypothesis. Proc. Natl. Acad. Sci. USA.

[35] Pelchen-Matthews A., Raposo G., Marsh M. (2004). Endosomes, exosomes and Trojan viruses. Trends Microbiol..

[36] Dzananovic E., Patel T.R., Deo S., McEleney K., Stetefeld J., McKenna S.A. (2013). Recognition of viral RNA stem-loops by the tandem double-stranded RNA binding domains of PKR. RNA.

[37] Narayanan A., Iordanskiy S., Das R., Van Duyne R., Santos S., Jaworski E., Guendel I., Sampey G., Dalby E., Iglesias-Ussel M. (2013). Exosomes derived from HIV-1-infected cells contain trans-Activation response element RNA. J. Biol. Chem..

[38] Nguyen T., Smith B.R., Tate M., Belz G., Barrios M.H., Elgass K.D., Weisman A.S., Baker P.J., Preston S., Whitehead L. (2017). SIDT2 Transports extracellular dsRNA into the cytoplasm for innate immune recognition. Immunity.

[39] Sampey G.C., Saifuddin M., Schwab A., Barclay R., Punya S., Chung M.-C., Hakami R.M., Zadeh M.A., Lepene B., Klase Z.A. (2016). Exosomes from HIV-1-infected cells stimulate production of pro-inflammatory cytokines through trans-activating response (TAR) RNA. J. Biol. Chem..

[40] Schlee M., Hartmann G. (2016). Discriminating self from non-self in nucleic acid sensing. Nat. Rev. Immunol..

