# Supplementary material for: Small Extracellular Vesicle Enrichment of a Retrotransposon-Derived Double-Stranded RNA: A Means to Avoid Autoinflammation?
Source: Biomedicines. 2021 Sep 1;9(9):1136. doi: 10.3390/biomedicines9091136 (PMC8472637; doi:10.3390/biomedicines9091136)
Supplement: Supplementary file 1 [file biomedicines-09-01136-s001.zip › biomedicines-1308893-SI.pdf]

SUPPLEMENTARY MATERIALS

Supplementary Figure S1. Overexpression of the motif-only construct in SH-SY5Y cells results in widespread cell death

Phase contrast light microscopy images of SH-SY5Y cells transduced with **(a)** motif alone and **(b)** full length VL30 constructs. Ten million cells were plated in T-175 flasks and allowed to grow with and without doxycycline for 48 hours. Left panels represent non doxycycline-treated cells, while right panels represent doxycycline-treated cells. Cells transduced with the motif only construct and treated with doxycycline did not proliferate and the majority died after 48 hours, as revealed by their lower cell density and dull appearance. In contrast, other cultures remained healthy after 48 hours.

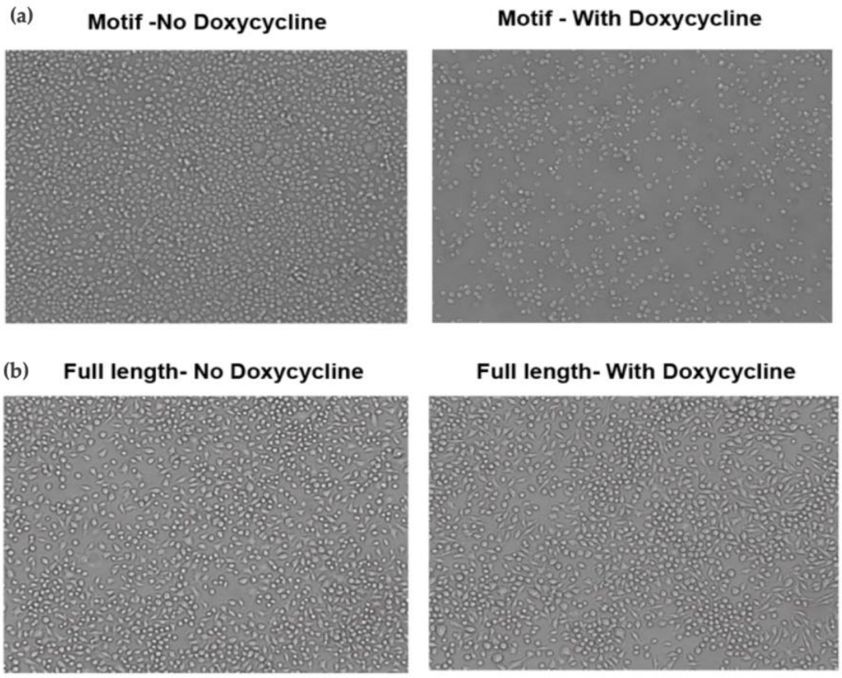

Supplementary Table S1A. Top 20 RNAs most enriched in exosomes

Rank	Gene Symbol	Gene Name	Fold Change	Adjusted P-value
1	A130040M12Rik	RIKEN cDNA A130040M12 gene (VL30 retroelement)	190.0	8.6E-09
2	Gm20186	predicted gene, 20186	44.0	5.9E-04
3	Gm29673	predicted gene, 29673	35.5	3.8E-03
4	Gm6993	predicted gene 6993	28.6	1.5E-04
5	Gm20269	predicted gene, 20269	28.2	3.7E-03
6	Myo18b	myosin XVIIIb	19.8	2.3E-04
7	Gm20752	predicted gene, 20752	16.0	1.1E-04
8	Cux2	cut-like homeobox 2	14.4	8.5E-05
9	Mafa	v-maf musculoaponeurotic fibrosarcoma oncogene family, protein A (avian)	13.8	2.4E-04
10	Rnf144a	ring finger protein 144A	13.1	9.5E-03
11	BC005512	cDNA sequence BC005512	12.2	2.1E-03
12	Slc17a7	solute carrier family 17 (sodium-dependent inorganic phosphate cotransporter), member 7	11.6	2.4E-04
13	Gm12505	predicted gene 12505	11.2	8.7E-05
14	Rap1gap2	RAP1 GTPase activating protein 2	11.0	4.6E-03
15	Metrn	meteorin, glial cell differentiation regulator	10.6	3.1E-04
16	Gm6139	predicted gene 6139	10.2	1.1E-04
17	Plekhh2	pleckstrin homology domain containing, family H (with MyTH4 domain) member 2	10.1	9.3E-04
18	Csf3	colony stimulating factor 3 (granulocyte)	9.9	9.9E-03
19	Lmo1	LIM domain only 1	9.8	9.7E-05
20	Cntn2	contactin 2	9.7	3.5E-04

Supplementary Table S1B. Top 20 RNAs most enriched in cells

No.	Gene Symbol	Gene Name	Log Fold Change	Adjusted P-value
1	Ndnf	neuron-derived neurotrophic factor	64.9	6.94E-07
2	Ighv6-3	immunoglobulin heavy variable 6-3	55.3	1.32E-06
3	Mir142	microRNA 142	49.5	8.63E-07
4	Tcaf2	TRPM8 channel-associated factor 2	46.2	1.60E-06
5	Scube1	signal peptide, CUB domain, EGF-like 1	44.6	6.77E-06
6	Flt3	FMS-like tyrosine kinase 3	44.3	7.31E-07
7	Malat1	metastasis associated lung adenocarcinoma transcript 1 (non-coding RNA)	43.4	1.48E-05
8	Tmtc2	transmembrane and tetratricopeptide repeat containing 2	42.5	8.94E-06
9	Scn4b	sodium channel, type IV, beta	41.4	3.20E-07
10	Spata31d1b	spermatogenesis associated 31 subfamily D, member 1B	37.8	6.82E-06
11	Il18r1	interleukin 18 receptor 1	37.8	2.99E-07
12	Nipal1	NIPA-like domain containing 1	37.5	6.66E-06
13	Kit	kit oncogene	36.3	8.96E-07
14	Ocln	occludin	35.5	1.69E-06
15	Slamf1	signaling lymphocytic activation molecule family member 1	34.5	1.85E-05
16	Sema7a	sema domain, immunoglobulin domain (Ig), and GPI membrane anchor, (semaphorin) 7A	34.3	2.09E-07
17	Tnni3	troponin I, cardiac 3	33.6	1.72E-06
18	Fosb	FBJ osteosarcoma oncogene B	33.1	1.18E-07
19	Adcy6	adenylate cyclase 6	32.9	2.91E-08
20	Ceacam15	carcinoembryonic antigen-related cell adhesion molecule 15	32.7	3.83E-08
